# Comparing the Performance of Automatic Milking Systems through Dynamic Testing Also Helps to Identify Potential Risk Factors for Mastitis

**DOI:** 10.3390/ani14192789

**Published:** 2024-09-26

**Authors:** Stefano Milanesi, Dario Donina, Viviana Chierici Guido, Francesca Zaghen, Valerio M. Sora, Alfonso Zecconi

**Affiliations:** 1 Associazione Regionale Allevatori della Lombardia, Via Kennedy 30, 26013 Crema, Italy; s.milanesi@aral.lom.it (S.M.); d.donina@aral.lom.it (D.D.); v.guido@aral.lom.it (V.C.G.); 2 One Health Unit, Department of Biomedical, Surgical and Dental Sciences, School of Medicine, University of Milan, Via Pascal 36, 20133 Milan, Italy; francesca.zaghen@unimi.it (F.Z.); valerio.sora@unimi.it (V.M.S.); 3 Department of Clinical and Community Sciences, School of Medicine, University of Milan, Via Celoria 22, 20133 Milan, Italy

**Keywords:** milking machine, automatic milking system, milking dynamic test, bimodality, mastitis

## Abstract

**Simple Summary:**

The performance of milking machines, whether in conventional or automated systems, can be evaluated using new-generation vacuum meters (dynamic testing). Access to data from these tests on automatic milking systems (AMSs) from various manufacturers and herds enabled the design of a retrospective study aimed at describing and comparing key milk emission parameters for different AMS brands, while also identifying potential mastitis risk factors. In total, 4878 individual quarter milkings were evaluated from cows in 48 different dairy herds. The findings revealed that factors such as milk yield and brand significantly influenced the variability of milking parameters. These results suggest that the interaction between AMSs and cows, along with the related milk emission physiology, plays a crucial role, similar to conventional milking. The observed differences in main milking parameters also correspond to parameters considered to predispose cows to mastitis. The most surprising result was the high frequency of two major mastitis risk factors (bimodality and irregular vacuum fluctuations).

**Abstract:**

Automatic milking systems (AMSs) are revolutionizing the dairy industry by boosting herd efficiency, primarily through an increased milk yield per cow and reduced labor costs. The performance of milking machines, whether traditional or automated, can be evaluated using advanced vacuum meters through dynamic testing. This process involves scrutinizing the system and milking routine to identify critical points, utilizing the VaDia™ logger (BioControl AS, Rakkestad, Norway). Vacuum recordings were downloaded and analyzed using the VaDia Suite™ software under the guidance of a milking specialist. Access to data from AMSs across various manufacturers and herds facilitated a retrospective study aimed at describing and comparing key milk emission parameters for different AMS brands while identifying potential mastitis risk factors. Using the proper statistical procedures of SPSS 29.1 (IBM Corp., Armonk, NY, USA), researchers analyzed data from 4878 individual quarter milkings from cows in 48 dairy herds. Results indicated a significant variability in milking parameters associated with quarter milk yield and AMS brand. Notably, despite AMSs standardizing teat preparation and stimulation, this study revealed a surprisingly high frequency of two major mastitis risk factors—bimodality and irregular vacuum fluctuations—occurring more frequently than in conventional milking systems. This study, one of the few comparing different AMS brands and their performance, highlights the crucial role of dynamic testing in evaluating AMS performance under real-world conditions.

## 1. Introduction

Automatic milking systems (AMSs) are significantly transforming the dairy sector and are either currently prevalent or are increasing in prevalence in many countries compared to traditional milking methods [1]. AMSs enhance herd efficiency, primarily through an increased yield per cow and reduced labor costs. Additionally, they positively impact both human and animal behavior and welfare [2,3,4,5].

This technology allows us to have a fully automatized milking process by udder quarter. Obviously, different manufacturers have developed different technologies to perform all the milking procedures, with potentially different outcomes on performances and udder health [1].

Milking represents one of the most important phases in milk production and therefore it is essential that the milker, the cow, and the machine are harmonized with each other. Indeed, the milking system can play a predisposing role in determining the development of mastitis, due to both its action on the teats and it being an active vehicle for the entry of pathogens into the udder [2,3,4]. On the other hand, the manual operations of the milking routine have a notable importance for the correct, quick, and efficient emission of milk, and for keeping an appropriate level of hygiene [5,6,7,8,9,10]. In conventional milking, the role of the individual milker, with regard to the milk emission, [11,12,13], is generally as strong as the role of the milking machine. One of the most important positive outcomes of the application of AMSs is the standardization of the milking procedure from cleaning, through stimulation to the post-milking teat disinfection. The standardization of the milking procedure is different based on the equipment and manufacturer, although all of them do not require a human intervention, thus dramatically reducing the potential negative effects of the milker. However, the effects related to the machine components and settings still exist when applying AMSs, and they may affect both milk quality and udder health [14,15].

The AMSs, like the conventional milking machine, interact with cows with different genetic, anatomical, physiological, and productive characteristics, with similar effects as conventional milking, although they are potentially of lower amplitude [14,16,17].

An increasing number of studies have focused on the application of AMSs since their commercial introduction in the 1990s, and most of these studies concern herd management, milk yield and quality, animal behavior, health and welfare, performance, and labor efficiency [2,4,6,7]. However, to the best of our knowledge, there is a gap in the current state of knowledge on the comparison between different AMSs of some aspects related to milk emission parameters (e.g., overmilking, vacuum fluctuations, bimodality) [18,19]. The availability of this knowledge may help in improving AMS performances and to take decisions on the type of technology to adopt, based on scientific data.

The performance of the milking machine during milking, in both conventional and automated systems, can be evaluated using new-generation flowmeters (dynamic testing). These procedures are currently applied to the dairy herds belonging to the Regional Association of Lombardy (ARAL) and to any dairy herd that requests this service. The availability of the data from these tests on AMSs of different manufacturers and herds allowed us to design a retrospective study aimed at describing and comparing the main parameters of milk emission for the different AMS manufacturers and the possible presence of factors associated with mastitis risk, as defined for conventional milking systems [5,20].

## 2. Materials and Methods

### 2.1. Herds and AMSs

All the 776 herds associated with the Regional Breeder Association of Lombardy (ARAL), having one or more AMS, and that received the dynamic testing during the year 2023, were considered. The characteristics of the AMSs present in Lombardy herds were summarized in Table 1. Among the six distinct brands, only four had at least 400 quarter dynamic test results; therefore, only these latter ones were furthermore considered in the statistical analysis.

### 2.2. Milking Dynamic Control

The Milking Control Service (SCM) provided by the ARAL includes periodic inspection of the milking system and its components, through mechanical tests and flow meter measurements, as required by the ISO 3918-5707-6690-UNI 11008 and following International Committee for Animal Recording guidelines (ICAR).

Dynamic control involves checking the system and the routine during milking, with the aim of identifying the critical points of the process and, with the analysis of the measurements and recorded data, providing indications and operational solutions to improve milking efficiency. The availability of a new portable digital vacuum logger (VaDia™; Biocontrol, Rakkestad, Norway) allows us to assess the milking process at the quarter level, an essential feature when AMSs are involved.

Indeed, the VaDia™ logger presents four vacuum recording channels, allowing us to record the vacuum dynamics in four distinct points of the milking unit. Vacuum recordings were performed continuously from unit attachment until the units were removed. All vacuum recordings were downloaded to a computer and analyzed with the VaDia Suite™ software (Biocontrol, Rakkestad, Norway). The graphic analysis of the vacuum recordings performed by the software under the supervision of the ARAL milking specialist allows us to identify several parameters, which are described in Table 2, following producers’ definitions.

Among the several parameters available, we selected the ones that, in our opinion, better describe milking performance: milking duration (MD; min); milk let-down (MLD, s); average milk flow (AMG; L/min); mean vacuum during milking (MVT, kPa); and mean vacuum at the peak (MVP, kPa). Moreover, parameters related to mastitis risks were also considered: overmilking (OMD, s); mean overmilking vacuum (MOV, kPa); mouthpiece chamber vacuum (MPC, kPa); delta vacuum fluctuations (DVFs, kPa; measured by the difference between the maximum and minimum vacuum level observed during the single milking); bimodality (BIM, N); and irregular vacuum fluctuations (IVF, N).

### 2.3. Data Recording and Statistical Analysis

Data were collected in a database with Excel 365™ (Microsoft, Redmond, WA, USA), and the statistical analyses were performed using the appropriate procedures of SPSS 29.0.1 (IBM Corp., Armonk, NY, USA).

Milk quality data were analyzed by a generalized linear model:

Y_jk_ = µ + B_j_ + M_k_ + B_j_ × M_k_ + e_jk_

where Y = dependent variables (milking parameters); µ = general mean; Bj = effect of brand (j = A, B, C, D); and Mk = effect of milk yield (j ≤ 2.5 kg/quarter; 2.6–3.5 kg/quarter; 3.6–4.5 kg/quarter; >4.5 kg/quarter).

The association between bimodality or irregular vacuum fluctuations with brand and milk yield was assessed using a binomial logistic regression model.

## 3. Results

### 3.1. Data Description

A total of 4878 dynamic tests of single quarter milkings were performed on cows from 48 different dairy herds. For the purpose of this study, only AMSs with at least 400 useful recordings were considered. Therefore, only four of the six different AMS brands available in Lombardy were considered.

The analysis of the frequency of the different brands by milk yield showed significant differences (Table 3). Brand A had a low frequency of records in low-yielding quarters, significantly different from the frequencies observed for all the other yield classes. The records in the highest yield class had the highest frequency among all the other classes. Brand B showed a linear distribution of the records among the yield classes, but the frequency of the lowest yield class was significantly higher than the other classes. Brand C showed a trend with a consistent and significant increase in the frequencies as milk yield increased. Brand D had the highest frequency of records in the <2.50 Kg class among all brands, and it was significantly different from all the other yield classes.

### 3.2. Main Milking Parameters

The results of the analysis of the influence of the brand, milk yield, and their interaction on the variance of the main milking parameters are reported in Table 4. The results showed that all the factors considered, except milk yield for MLD, had a significant influence on the variability of the factors considered. The models showed a R^2^ in the range of 20–30%, except for MLD, suggesting, as expected, an important influence of AMSs on the milking emission curves.

**Figure 1:**
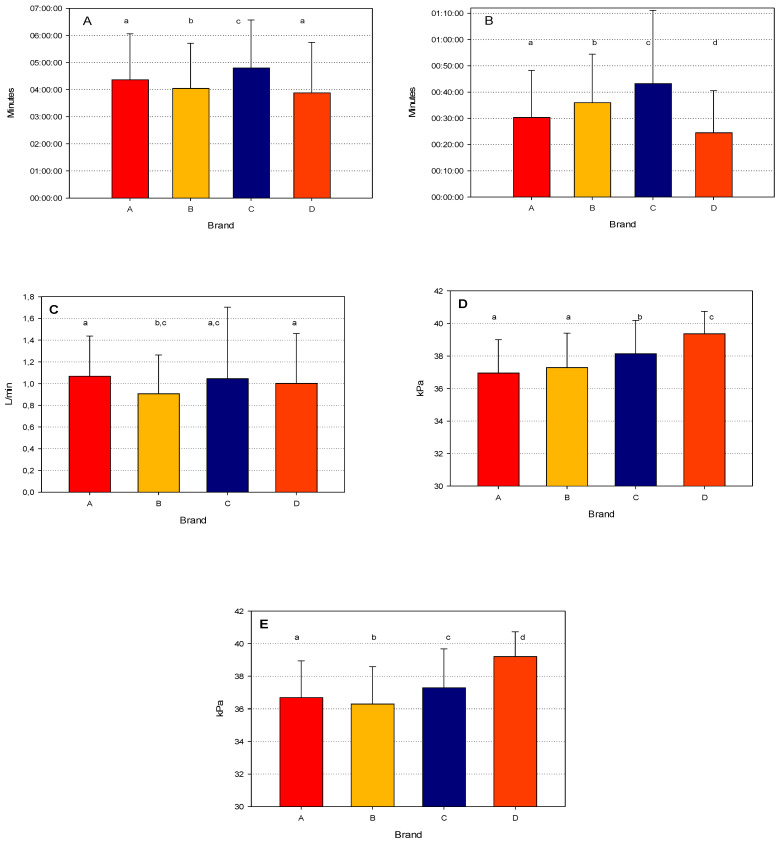
Mean values (±std.dev.) for the milking parameters ((**A**): milking duration; (**B**): milk let-down; (**C**): average milk flow; (**D**): mean vacuum during milking; and (**E**) mean vacuum at peak) for the four brands considered. Within each figure, means (bars) with different letters (a–d) are statistically different (α = 0.05).

Figure 1 describes the mean values of the five factors considered classified by brand. The means for MLD and for MVP were always significantly different among brands, with significant differences mainly between brand C and D when compared to the other two brands. For all the other parameters, the differences among the brands were numerically less evident, but it should be noted that the MD was significantly longer for brand C, which is probably associated with the longer MLD time.

The GLM analysis of the influence of the interaction of milk yield and brand was reported in Table 5. When considering the MD, as expected, an increasing trend was observed across milk yield and among all the brands, but significant differences were also observed among the brands within each yield class. In fact, brand D had the shortest MD among all the brands and yields. Similarly, the MLD time was shorter for brand D, but significant differences among brands were consistent only in the highest milk yield class. It should be noted that the MLD time was shorter for brands A and D in the lowest yield classes, with an increasing trend for brand A, but not any other brands. This was confirmed by the mean MLD values in the different milk yield classes, which showed that the increasing trend in the mean values was not as great as for the MD. These results suggest that the interaction of the AMS with the cows and the teat-stimulation process play an important role, as also observed in conventional milking [21].

As expected, the average milk flow increased with increasing milk yield. Statistical differences were observed mainly in the highest yield classes, with the highest means for brands A and D. Although brands C had the longest MD and MLD, they also had the highest AMF, suggesting that a longer milking process, in this case, does not affect AMF.

The mean vacuums during milking and at peak flow were both in the acceptable range of 36.4–39.5 kPa. However, there were significant differences among brands and yield. Brand D had the highest values for both measurements, with significant differences from the other brands. This result, which is associated with the shorter MD and MLD observed, suggests that the higher vacuum applied in these AMSs results in a shortening of MD and MLD.

### 3.3. Parameters Associated with Mastitis Risk

The analysis of the factors influencing the variability of the four parameters that are considered as potential mastitis risk factors were summarized in Table 6. A brand and its interaction with milk yield were always statistically significant and the corresponding GLM models showed a relatively high R^2^ for OMD and for DVFs.

**Figure 2:**
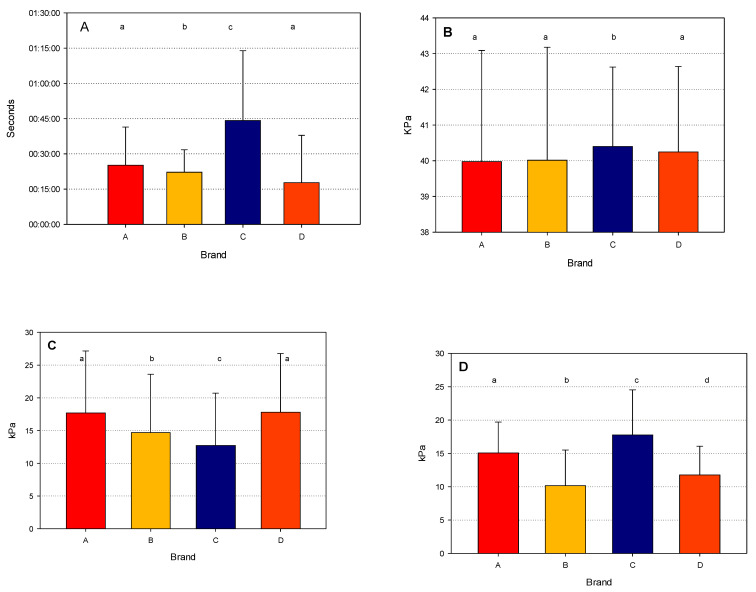
Mean values (±std.dev.) for the milking parameters ((**A**): overmilking; (**B**): mean overmilking vacuum; (**C**): mouthpiece chamber vacuum; and (**D**): delta vacuum fluctuations) for the four brands considered. Within each figure, means (bars) with different letters (a–d) are statistically different (α = 0.05).

The OMD was significantly higher for brand C (Figure 2A), while it was very similar for the other brands, while the MOV was around 40 kPa for all the four brands, although a significantly higher mean value was observed for brand C (Figure 2B).

The AMS of the same brand showed a significantly lower vacuum level in the MPC (Figure 2C), while the DVFs (Figure 2D) were significantly different among all the brands, with the lowest mean value for brand B and the highest for brand C.

The analysis of the interactions between brand and milk yield for the four parameters are reported in Table 7. The OMD was significantly longer in the higher yield class. This result is probably influenced by the values observed for the brand C AMS, which had values almost double of those of the other brands. On the other hand, the brand D AMS showed the shortest mean values, with a decreasing trend with increasing milk yield. This pattern was not observed for any of the other brands.

The mean vacuum during overmilking was not overly different among brands and milk yield classes, although some statistically significant differences can be observed. On the contrary, the MPCs were relatively similar among milk yield classes, but differed among brands, being particularly low for the brand C AMS. In addition, the MPCs were higher for brands A and D when compared to the other brands.

The greatest variation was observed for the DVFs, which were significantly different among brands and for all the yield classes. An increasing trend was observed among the milk yield classes, with the highest level in the >4.5 Kg class. Overall, the lowest values were observed for brands B and D, with mean values significantly lower than those observed for the brand A and C AMSs.

### 3.4. Frequencies of Bimodality and Irregular Vacuum Fluctuations

The VaDia™ tools applied during milking also allow the assessment of two of the main risk factors for mastitis: bimodality and irregular vacuum fluctuations [22,23,24,25].

**Figure 3:**
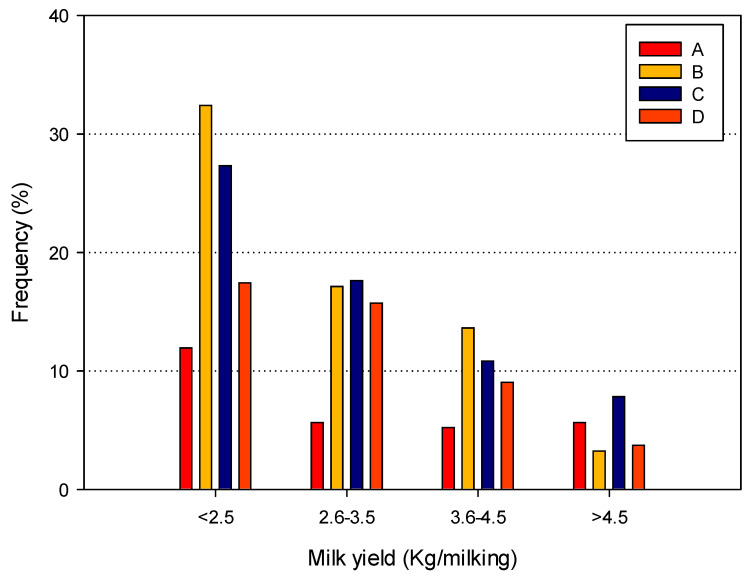
Mean frequency of bimodality for the four yield classes and the four AMS brands considered.

The patterns of BIM among brands and milk yield classes were shown in Figure 3, while Table 8 reports the results of the logistic regression analysis estimating the odds ratios for BIM based on brand and yield.

The overall trend showed that, as expected, the frequency of BIM decreases with an increasing milk yield, and this observation was confirmed by statistical analysis with a significant protective (values < 1) odds ratio when compared to the frequency of BIM with the <2.5 kg class. Large differences in the frequency of bimodality were observed among brands, with apparently lower values for brand A when compared to the other brands. However, statistical analysis did not confirm this observation.

Indeed, only in the case of brand D, a significant odds ratio was observed and its value < 1 indicates that the risk of BIM for this latter brand is reduced when compared to brand A. This result supports previous observations on MLD, being the shortest among all the brands for all the milk yield classes.

**Figure 4:**
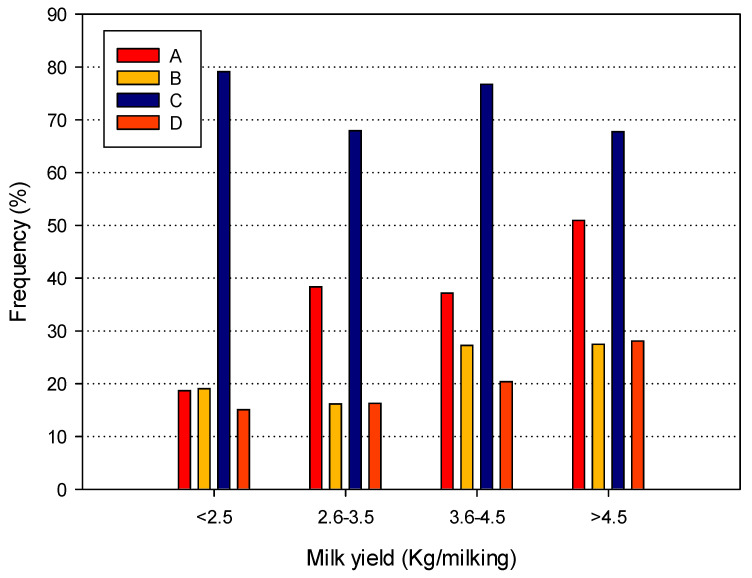
Mean frequency of irregular vacuum fluctuations for the four yield classes and the four AMS brands considered.

The same analysis applied to IVF showed that the AMS of brand C had the highest frequency, with values close to 80%, while in all the other cases the values were around or below 40% (Figure 4). The statistical analysis, reported in Table 9, showed that the odds ratio was <1 for all the yield classes > 2.5 Kg, confirming previous observations on BIM. When the brands were considered, the results confirmed that the odds ratio for IVF was very high and significant for brand C, when compared to brand A, while it was below 1 when brands B and C were compared to brand A. This different pattern may be explained by previous observations on MLD, OMD, and DVF values, which were always significantly higher for the brand C AMS.

## 4. Discussion

The increasing use of AMSs in many countries represents a true revolution in the dairy industry, with positive implications for cows, farmers, and, more generally, the sustainability of dairy herds [8,26]. These tools have standardized several aspects of milking, avoiding problems related to the behavior and performance of the milker, but not those related to the interaction between the cow and the milking machine. In addition, the performance of AMSs from different manufacturers may differ and should be considered when adopting this technology.

The evaluation of the performance of a milking machine, whether automated or conventional, has improved in recent years thanks to the availability of new electronic vacuum meters that allow the evaluation of the milking of individual quarters for many different parameters, which is crucial when AMSs are involved.

The availability of these new test tools, and in particular VaDia™, within a standardized evaluation procedure, makes it possible to describe the performance of a milking machine at the quarter level, to identify the presence of potential risk factors for milk quality and udder health, and to compare the different machines [27].

The analysis of nearly 5000 milkings from four different AMSs used in 48 Italian dairy herds has undoubtedly revealed new and, in some cases, unexpected results. One of these results showed that milking performance is influenced by the milk yield of each quarter. As such, this result was expected based on previous studies [14,28], and the knowledge based on conventional milking [16,17]. However, the significant interaction of milk yield classes with the four different AMS brands suggests that the expected milk yield per milking is a variable that needs to be considered when choosing which technology to purchase or when defining the number of milkings allowed daily for each cow. In fact, a lower milk yield/milking is associated with higher MVT and MVP levels and AMG, but also with a higher frequency of BIM. The latter condition is one of the best-known risk factors for teat injuries and mastitis [27,29,30].

The differences among manufacturers were also statistically significant when MLD and, consequently, the MD were considered. These differences were found to be large among AMSs and could affect both the efficiency of the whole milking, delaying cow trafficking, but also cow welfare, leaving several cows standing in waiting for a free AMS [31]. The difference among AMS brands in MLD may be explained by the different systems of quarter pre-milking preparation, milking vacuum, and by the teat cup detachment setting [32,33,34], but also by other factors such as pulsation rates and liner characteristics, which were not considered in this study.

The differences observed in the main milking parameters were also reflected in the parameters considered to predispose cows to the development of mastitis. Overmilking was observed in all AMSs, although with different amplitudes and with significant differences between manufacturers, although milking is independent for each quarter. This result was unexpected because the teats are milked separately, reducing the effect of conventional milking where cluster detachment is based on the total milk flow from all the four quarters to keep the cluster on [35]. The differences among brands were also unexpected, but they are supported by the different values of the milking parameters (e.g., MD, MLD, DVF) that were correlated with the OMD.

However, the most unexpected result was the presence of high frequencies of two important factors for mastitis risk (bimodality and irregular vacuum fluctuations) [5,27]. The absence of human intervention during milking, the standardized milking procedure, and the presence of a stimulation phase, albeit with different methods, led us to expect that these two factors should be observed with very low frequencies. On the contrary, bimodality was observed with a frequency ranging from 5 to 32%, and three out of four brands showed frequencies > 15%, a level considered as critical [27,29], when milk yield was <3.5 Kg/milking. In fact, a decrease in odds ratio values was observed with an increasing milk yield. This result supports the previous observation on the need to consider the quarter milk yield as a critical factor and suggests that the individual milking frequency should be defined based on the expected yield, which should be at least >3.5 Kg/milking, on quarter bases.

The pattern for IVF was even worse, with frequencies ranging from 18 to 79%. In this case, the frequencies and the odds ratios decreased as the yield increased. Moreover, the AMS brand showed a greater role compared to BIM, with one of the brands being significantly associated with the presence of IVF. It should also be noted that the overall frequencies of IVF were higher when compared to conventional milking systems, probably because AMSs have a more complex milking system, which may introduce additional points of potential vacuum instability.

This study is one of the few that compares different AMS brands and their milking performance using dynamic testing under real-life conditions. It highlights the importance of such testing for evaluating AMS performance in the field and indicates that both AMSs and conventional milking systems may pose risks for teat damage and mastitis. This study has the limitation of being a point assessment of the AMS performance and does not consider the long-term effects on cow health. Therefore, this study should be complemented with follow-up research to further investigate the relationship between milking parameters observed during dynamic testing and the incidence of mastitis or teat injuries.

## 5. Conclusions

Automatic milking systems are increasingly replacing conventional milking methods. These systems offer several significant advantages, including enhanced milk production, improved human and animal welfare, and, potentially, overall herd sustainability. The results of this study indicate substantial differences among various AMSs, suggesting that comparisons should be based on field data collected with dynamic testing. Findings also revealed that AMSs do not always resolve certain milking process issues linked to mastitis risk, such as overmilking and bimodality, which are commonly observed in conventional milking systems. Although numerous studies have been conducted on AMSs, there remains a need for dynamic testing under field conditions to evaluate their performance and identify issues that can be resolved through machine fine-tuning, thus mitigating the risk of teat impairment and mastitis.

## Figures and Tables

**Table 1:** Characteristics of the automatic milking systems applied in Lombardy dairy herds modified from [1].

Characteristics	Brand					
	A	B	C	D	E ^1^	F ^1^
**Traffic**	Forced/free	Free	Forced/free	Free	Free	Free
**Milking boxes**	Single	Single	Single/multi	Single	Single/multi	Single/multi
**Robot arm type**	Special	Special	Special	Special	Special	Industrial
**Drive control of the robot arm**	Hydraulic	Electric	Electric	Pneumatic/electric	Electric	Electric
**Robot arm location**	Side of the udder	Side of the udder	Side of the udder	Side of the udder	Behind the udder	Side of the udder
**Teat cleaning**	Special cluster	Rotating brushes	Milking cluster	Rotating brushes	Special cluster	Special cluster
**Entry and exit from milking box**	Combined	Combined	Combined	Straight	Combined	Combined

^1^ Not considered furthermore due to the small number of milkings available.

**Table 2:** Milking parameters measured by VaDia™ during milking.

**Teat-end vacuum**	Average teat-end vacuum with milk flow
	Vacuum level and fluctuations
	Teat-end vacuum during peak flow
**Milking time testing**	Presence of bimodal milking
	Overmilking duration and vacuum
	Automatic take-off functioning
	Liner fit for teats
**Pulsator testing**	Correct timing of the A, B, C, and D phases
	Vacuum build-up in pulsator channels
**Cluster falloff testing**	Vacuum recovery according to the ISO 6690 standard ISO 6690:2007 (Milking machine installations—Mechanical tests)
**Slug test**	Physical aspect of the slug in the milk line
	Air injection rate and volume of water

**Table 3:** Description of the frequency of individual milking records by brand and milk yield recorded during the assessment.

Brand	Units	Frequency by Milk Yield (kg/Quarter)				
		<2.50	2.51–3.50	3.51–4.50	>4.50	Total AMS Frequency
**A**	N	59 ^a,1^	107 ^b^	116 ^b^	216 ^c^	498
	%	11.8%	21.5%	23.3%	43.4%	10.2%
**B**	N	247 ^a^	205 ^b^	169 ^b,c^	157 ^c^	778
	%	31.7%	26.3%	21.7%	20.2%	15.9%
**C**	N	110 ^a^	262 ^b^	288 ^b,c^	334 ^c^	994
	%	11.1%	26.4%	29.0%	33.6%	20.4%
**D**	N	769 ^a^	661 ^b^	631 ^b^	547 ^c^	2608
	%	29.5%	25.3%	24.2%	21.0%	53.5%
**Total**	N	1185	1235	1204	1254	4878
	%	24.3%	25.3%	24.7%	25.7%	100%

^1^ Values within each brand with different letters are statistically different at χ^2^ test (α = 0.05).

**Table 4:** Statistically significant factors estimated by general linear model statistical analyses affecting the milking parameters considered. A star indicates the presence of a statistically significant effect of the factor on the parameter.

Parameter	Factors				
	Brand	Milk Yield	Brand × Milk Yield	*p* (Model)	R^2^
**Milking duration**	*	*	*	<0.001	22.1%
**Milk let-down**	*	n.s. ^a^	*	<0.001	13.7%
**Average milk flow**	*	*	*	<0.001	20.2%
**Mean vacuum during milking**	*	*	*	<0.001	25.1%
**Mean vacuum at peak**	*	*	*	<0.001	29.7%

^a^ not significant (α = 0.05).

**Table 5:** Statistically significant factors estimated by general linear model statistical analyses affecting the milking parameters considered.

Parameter	Brand	Milk Yield (kg/Quarter)			
		<2.50	2.51–3.50	3.51–4.50	>4.50
**Milking duration** **(min)**	A	2:47:55 ^a,1^	3:43:47 ^a^	4:08:17 ^a,c^	5:14:40 ^a^
	B	3:05:53 ^a,c^	3:39:47 ^a^	4:24:56 ^a,b^	5:39:48 ^b^
	C	3:24:31 ^c^	4:25:42 ^b^	4:35:32 ^b^	5:45:03 ^b^
	D	3:05:50 ^a,c^	3:39:35 ^a^	3:59:51 ^c^	5:09:01 ^a^
	Mean	3:06:44	3:45:46	4:12:43	5:23:26
**Milk let-down** **(sec)**	A	0:25:56 ^a^	0:27:52 ^a^	0:33:04 ^a^	0:31:25 ^a^
	B	0:40:35 ^b^	0:33:47 ^b^	0:32:43 ^a^	0:35:28 ^b^
	C	0:45:01 ^c^	0:42:37 ^c^	0:44:27 ^b^	0:42:20 ^c^
	D	0:23:46 ^a^	0:25:32 ^a^	0:24:27 ^c^	0:24:45 ^d^
	Mean	29:21	30:44	31:13	31:55
**Average milk flow** **(l/min)**	A	0.76 ^a,b^	0.91 ^a^	1.09 ^a,c^	1.22 ^a^
	B	0.73 ^b^	0.90 ^a^	0.97 ^a,b^	1.10 ^b^
	C	0.63 ^a^	0.78 ^b^	0.95 ^b^	1.47 ^c^
	D	0.74 ^a,b^	0.97 ^a^	1.16 ^c^	1.23 ^a^
	Mean	0.73	0.91	1.08	1.28
**Mean vacuum during milking** **(kPa)**	A	37.45 ^a^	37.49 ^a^	37.17 ^a^	36.43 ^a^
	B	37.82 ^a^	37.26 ^a^	36.86 ^a^	36.98 ^b^
	C	38.98 ^b^	38.22 ^b^	38.06 ^b^	37.83 ^c^
	D	39.55 ^c^	39.44 ^c^	39.32 ^c^	39.02 ^d^
	Mean	39.03	37.65	38.47	37.99
**Mean vacuum at peak** **(kPa)**	A	39.02 ^a^	37.30 ^a^	36.92 ^a^	36.18 ^a^
	B	36.55 ^a^	36.21 ^b^	35.99 ^b^	36.34 ^a^
	C	38.03 ^b^	37.38 ^a^	37.15 ^a^	37.11 ^b^
	D	39.33 ^c^	39.31 ^c^	39.22 ^c^	38.92 ^c^
	Mean	38.51	38.21	38.05	37.64

^1^ Values within each column with different letters are statistically different (α = 0.05).

**Table 6:** Statistically significant factors estimated by general linear model statistical analyses affecting the milking parameters related to mastitis risk. A star indicates the presence of a statistically significant effect of the factor on the parameter.

Parameter	Factors				
	Brand	Milk Yield	Brand × Milk Yield	*p* (Model)	R^2^
**Overmilking**	*	n.s. ^a^	*	<0.0001	19.8%
**Mean overmilking vacuum**	*	*	*	0.013	1.3%
**Mouthpiece chamber vacuum**	*	n.s.	*	<0.001	5.8%
**Delta vacuum fluctuations**	*	*	*	<0.001	24.8%

^a^ not significant (α = 0.05).

**Table 7:** Statistically significant factors estimated by general linear model statistical analyses affecting the milking parameters related to mastitis risk.

Parameter	Brand	Milk Yield (kg/Quarter)			
		<2.50	2.51–3.50	3.51–4.50	>4.50
**Overmilking (sec)**	A	28:41 ^a,1^	25:05 ^a^	25:51 ^a^	23:50 ^a^
	B	21:25 ^b^	22:05 ^a^	23:27 ^a^	22:16 ^a^
	C	41:21 ^c^	41:54 ^b^	43:23 ^b^	47:45 ^b^
	D	20:52 ^b^	16:16 ^c^	16:02 ^c^	16:59 ^c^
	Mean	23:16	23:26	24:34	27:01
**Mean overmilking vacuum (kPa)**	A	40.44 ^a^	40.62 ^a,c^	39.67 ^a^	39.70 ^a^
	B	39.98 ^a^	40.16 ^a^	40.18 ^a,b^	39.69 ^a^
	C	40.51 ^a^	40.37 ^a,c^	40.49 ^b^	40.30 ^b^
	D	40.62 ^a^	40.39 ^c^	40.09 ^a^	39.70 ^a^
	Mean	40.47	40.37	40.15	39.86
**Mouthpiece chamber vacuum (kPa)**	A	16.47 ^a,b^	16.82 ^a,b^	17.87 ^a^	18.39 ^a^
	B	15.92 ^b^	15.04 ^b^	14.62 ^b^	12.41 ^b^
	C	12.96 ^c^	12.09 ^c^	11.90 ^c^	13.85 ^b^
	D	17.56 ^a^	17.94 ^a^	17.25 ^a^	18.68 ^a^
	Mean	16.74	16.12	15.66	16.56
**Delta vacuum fluctuations (kPa)**	A	13.80 ^a^	14.87 ^a^	14.11 ^a^	16.02 ^a^
	B	8.41 ^b^	10.62 ^b^	11.29 ^b^	11.10 ^b^
	C	17.22 ^c^	17.29 ^c^	19.31 ^c^	17.02 ^c^
	D	10.73 ^d^	11.51 ^d^	12.22 ^d^	12.95 ^d^
	Mean	11.00	12.88	13.96	14.33

^1^ Values within each column with different letters are statistically different (α = 0.05).

**Table 8:** Results of logistic regression analysis of the association between bimodality and the risk factors: brand and milk yield.

					95% C.I. Odds Ratio	
Factors	B	S.E.	Sign	Odds Ratio	Lower	Higher
**Brand**			<0.001			
**B vs. A**	−0.046	0.156	n.s. ^a^	0.96	0.70	1.30
**C vs. A**	0.178	0.156	n.s.	1.19	0.88	1.62
**D vs. A**	−0.864	0.131	<0.001	0.42	0.33	0.55
**Milk yield (kg/Quarter)**			<0.001			
**2.6–3.5 vs. <2.5**	−1.525	0.082	<0.001	0.22	0.18	0.26
**3.6–4.5 vs. <2.5**	−2.086	0.100	<0.001	0.12	0.10	0.15
**>4.5 vs. <2.5**	−2.045	0.141	<0.001	0.05	0.04	0.06

^a^ not significant (α = 0.05).

**Table 9:** Results of logistic regression analysis of the association between irregular vacuum fluctuations and the risk factors: brand and milk yield.

					95% C.I. Odds Ratio	
Factors	B	S.E.	Sign	Odds Ratio	Lower	Higher
**Brand**			<0.001			
**B vs. A**	−1.007	0.122	<0.001	0.37	0.29	0.464
**C vs. A**	1.223	0.117	<0.001	3.40	2.70	4.27
**D vs. A**	−1.323	0.098	<0.001	0.27	0.22	0.32
**Milk yield (kg/Quarter)**			<0.001			
**2.6–3.5 vs. <2.5**	−0.709	0.073	<0.001	0.49	0.43	0.57
**3.6–4.5 vs. <2.5**	−0372	0.070	<0.001	0.39	0.60	0.79
**>4.5 vs. <2.5**	−0.239	0.066	<0.001	0.79	0.69	0.90

## Data Availability

Data are unavailable due to privacy restrictions.
